# Application of ADSCs and their Exosomes in Scar Prevention

**DOI:** 10.1007/s12015-021-10252-5

**Published:** 2021-09-12

**Authors:** Cong Li, Shuqiang Wei, Quanchen Xu, Yu Sun, Xuchao Ning, Zhiguo Wang

**Affiliations:** 1 The Affiliated Hospital of Qingdao University, Qingdao University, Qingdao, 266021 Shan Dong People’s Republic of China; 2 The Second Affiliated Hospital of Qingdao University (Qingdao Central Hospital), Qingdao, 266021 Shan Dong People’s Republic of China; 3 Department of Burn and Plastic Surgery, the Affiliated Hospital of Qingdao University, Qingdao, 266021 Shan Dong People’s Republic of China

**Keywords:** Adipose tissue-derived stem cells, Exosomes, Fibroblasts, Myofibroblasts, Wound healing, Scar

## Abstract

Scar is a common way of healing after tissue injury. The poor scar healing will not only cause dysfunction of tissues and organs but also affect the appearance of the patients’ body surface, which causes the pressure of life and spirit to the patients. However, the formation of scar tissue is an extremely complex process and its mechanism is not fully understood. At present, there is no treatment method to eliminate scars completely. Fibroblasts are the most abundant cells in the dermis, which have the ability to synthesize and remodel extracellular matrix (ECM). Myofibroblasts actively participate in the wound healing process and influence the outcome. Therefore, both of them play important roles in wound healing and scar formation. Adipose tissue-derived stem cells (ADSCs) are pluripotent stem cells that can act on target cells by paracrine. Adipose tissue stem cell-derived exosomes (ADSC-Exos) are important secretory substances of ADSCs. They are nanomembrane vesicles that can transport a variety of cellular components and fuse with target cells. In this review, we will discuss the effects of ADSCs and ADSC-Exos on the behavior of fibroblasts and myofibroblasts during wound healing and scarring stage in combination with recent studies.

**Graphical Abstract:**

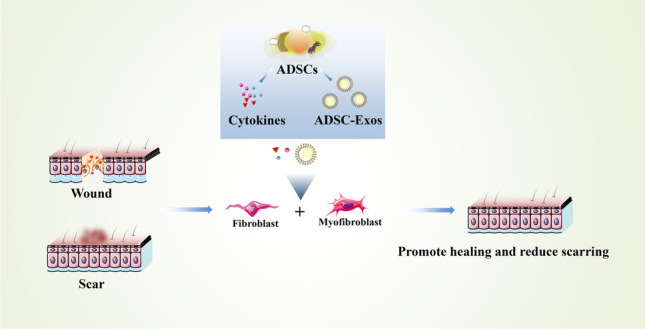

## Introduction

Scar formation is related to individual race, gender, age, as well as wound tension, location, and injury pattern [1]. Besides, the forming of an abnormal scar is due to severe inflammatory response, poor blood supply, and the imbalance of fibroblasts, keratinocytes, cytokines, etc., which lead to excessive deposition of ECM in the process of wound healing [2–5]. Billions of dollars are spent every year to treat wounds and scars [6], even severe scars can cause mental problems [7].

Fibroblasts play an important role in wound healing and scar formation [8], which are essential for ECM production, but overproduction may be detrimental to the outcome of scarring [9]. Myofibroblasts are considered to be the main fibrogenic cells in wound healing [10]. The regulation of some signaling pathways in fibroblasts and myofibroblasts is beneficial to reduce scar formation. For example, angiotensin-converting enzyme inhibitors (ACEI) have shown anti-fibrotic properties in scar formation by inhibiting the TGF-β1/Smad and TGF-β1/TAK1 signaling pathways [11], and rapamycin inhibits PI3K/Akt/mTOR pathway to promote cell apoptosis and reduce keloid activity [12]. The reprogramming of myofibroblasts into other cells in scar tissue is also a potential treatment. Plikus et al. demonstrated that myofibroblasts can be transformed into adipocytes under the action of BMP4 [13]. Therefore, the regulation of fibroblasts and myofibroblasts is a very important target in scar treatment.

Considerable research efforts have indicated that stem cell therapy is effective and promising for many diseases that cannot be treated with traditional methods [14]. Recent tissue engineering and cell therapy strategies have demonstrated the significance of ADSCs in regenerative medicine [15–19]. In terms of wound healing, ADSCs showed positive impact in promoting wound healing and scar treatment [20]. For example, ADSCs can accelerate the healing of diabetic wound via the recruitment and differentiation of endothelial progenitor cells [21]. Specifically, ADSCs can play an important role in wound healing and scar formation by reducing inflammation, promoting angiogenesis, reducing apoptosis, transporting mitochondria, and secreting exosomes in damaged tissues [17, 22].

ADSC-Exos are derived from ADSCs, so they have similar effects and also have positive impact in wound healing and scar treatment. Hu et al. found that mice wounds with adipose tissue healed more quickly and efficiently. Besides, CD63, an exosomal specific marker, was more expressed [23]. In the process of wound healing, many studies have shown that exosomes play a strong part in angiogenesis, immune regulation, and the reduction of ischemia-reperfusion injury [24].

It is very important for patients to reduce scar formation after skin injury. Fibroblasts and myofibroblasts perform a decisive role in wound healing outcomes. So, based on current research, the purpose of this review is to discuss the effect of ADSCs and ADSC-Exos on reducing scar formation by regulating the behavior of fibroblasts and myofibroblasts.

## Scar Formation Process

### From the Wound to the Scar

Skin wound healing mainly includes four stages: inflammatory response, cell proliferation, migration, and ECM remodeling [7]. The initial inflammatory phase prevents blood loss, infection and clears debris, while the subsequent proliferative phase supports keratinocyte proliferation and migration to reseal the epithelium. In the remodeling phase, adipocytes, fibroblasts, and ECM fill the wound area to form scars [25]. In normal wound healing, the fibrin clot releases chemokines and initiates the migration of white blood cells to the injured area. Neutrophils are the first cells to enter the wound tissue in the early stages of inflammation. Macrophages replace neutrophils in the late inflammatory stage. During abnormal wound healing, a large number of macrophages release cytokines inappropriately between the late inflammatory stage and the proliferative stage, which promote the formation of the pathological scar. In remodeling phase, new ECM molecules, such as fibulin, Type III collagen (Col-III), and Col-I, are deposited sequentially. Collagen remodeling gradually increases the strength of scar tissue and reaches a plateau about 7 weeks after trauma [5]. Many factors determine the complexity and diversity of scar formation [2].

When skin and blood vessels are damaged, a temporary matrix, made up mainly of fibrin, triggers an inflammatory response. Fibroblasts migrate to wound surface, where they acquire myofibroblast phenotypes and contribute to granulation tissue formation. ECM components are synthesized and deposited by myofibroblast, which gradually replace the temporary matrix. In the later remodeling process, the synthesis of ECM is greatly reduced, and Col-I replaces Col-III. Finally, the apoptosis of fibroblasts and blood vessel cells greatly reduce the cellular component of scar tissue [26]. Dermal fibroblasts produce elastin and fibrin, eventually forming elastic fibers, which then give the skin some elasticity and participate in the recovery of dermal structure [27]. Meanwhile, the biological behavior of skin fibroblasts are affected by the skin tension in the process of scarring. Studies have shown that skin fibroblasts show stronger hypertrophic scar changes at 10–15% stretch [28, 29].

Furthermore, scar formation has a relationship with EMT. EMT refers to the biological process in which different types of epithelial cells are transformed into mesenchymal cells through a series of biological changes under the influence of different factors. EMT is necessary for normal re-epithelialization and ECM deposition: the continued and uncontrolled transformation from epithelial cells to fibroblasts and myofibroblasts may result in pathological scar. In the process of EMT, pseudopodia appear in the front end of cuboidal keratinocytes and the cells transform into a spindle shape, which promote cell migration. After epithelialization, keratinocytes restore epithelial phenotype and reestablish tight cell connection and barrier functions. Simultaneously, EMT-derived myofibroblasts contract and secrete ECM during the early stages of skin wound healing. In the later stage, unresolved inflammation can affect EMT and lead to abnomal scar [7].

### The Characteristics of Pathological Scar

Excessive scar is thought to be the result of the accumulation of inflammatory cells and fibroblasts in wound areas. Scars can be divided into two types according to the color, texture, and patients’ feeling: immature and mature. Scars can be classified as HTS, keloids, atrophic scars, and scar cancer on the basis of anatomy [2]. Pathological scar mainly refers to HTS and keloid. HTS is defined as abnormal deposition and remodeling of ECM, which is usually caused by skin lesions (trauma, deep burns, and surgery) [30]. The most prominent feature of HTS is the differentiation of fibroblasts into myofibroblasts [30], which is controlled by the changed chemical and mechanical microenvironment of the repaired tissue [31]. In HTS and keloids, excessive ECM accumulation is caused by fibroblast proliferation, apoptosis and its subsequent imbalance of protein products. In keloids, fibroblast proliferation is more pronounced and resistant to FAS-mediated apoptosis [5]. HTS, which usually occurs in areas where the skin has been stretched, grows rapidly from 4 to 12 weeks before flattening and subsiding over time. However, keloids protrude from the wound site and grow invasiely, rarely subsiding [32]. In keloids, there is a severe inflammatory response and fibroblasts show high sensitivity to TGF-1. In HTS, the ratio between Col-I and Col-III (6:1) is lower than in keloids (17:1), but the ratio in normal skin is 5:1 [32].

## Characteristics of Fibroblasts and Myofibroblasts and their Role in Scar Formation

### Fibroblasts

Pedigree analysis experiments in mice and chickens showed that embryonic dermal fibroblasts originated in different parts of the embryo [33]. Dermal fibroblasts are a heterogeneous population of cells whose specificity depends mainly on their position relative to the layers of the dermis [34]. Papillary dermal progenitor cells give rise to papillary dermal fibroblasts (PF) and dermal papilla (DP), while reticular dermal fibroblast progenitor cells give rise to reticular fibroblasts (RF) and dermal white adipose tissue (DWAT). PF and DP are involved in hair follicle morphogenesis and follicle cycle [33]. The papillary layer has more fibroblasts with high enzyme activity than the reticular layer [34]. The ability to synthesize Type I collagen (Col-I) is a major characteristic of fibroblasts. Fibroblasts contribute to the synthesis and remodeling of ECM, and its remodeling function is mainly realized by the synthesis of metalloproteinases and metalloproteinase inhibitors. The density change of dermal fibroblasts in vitro shows that the critical density of dermal fibroblasts is necessary for the formation of self-tissue matrix [35]. Fibroblasts are highly expressive of fibrogenic markers (CD90, PDGFR-α, PDGFR-β, leucine-rich small proteoglycans, decorin, and lumican). Functional fibroblasts do not express α-smooth muscle actin (α-SMA) [36]. The migration of fibroblasts to the wound is regulated by inflammatory mediators, in which the chemokine CCL-2 enables fibroblasts to be recruited to the wound site and differentiate into myofibroblasts [37]. Lack of mature fat cells in the skin or inhibition of fat formation prevents fibroblasts from being collected at the wound site, leading to delayed wound closure [33]. A large number of studies have demonstrated that soluble physiological factors such as IL-1, TNF, TGF-β1, IL-13 and connective tissue growth factor (CTGF) are related to fibroblast proliferation and differentiation [37].

### Myofibroblasts

**Fig. 1:**
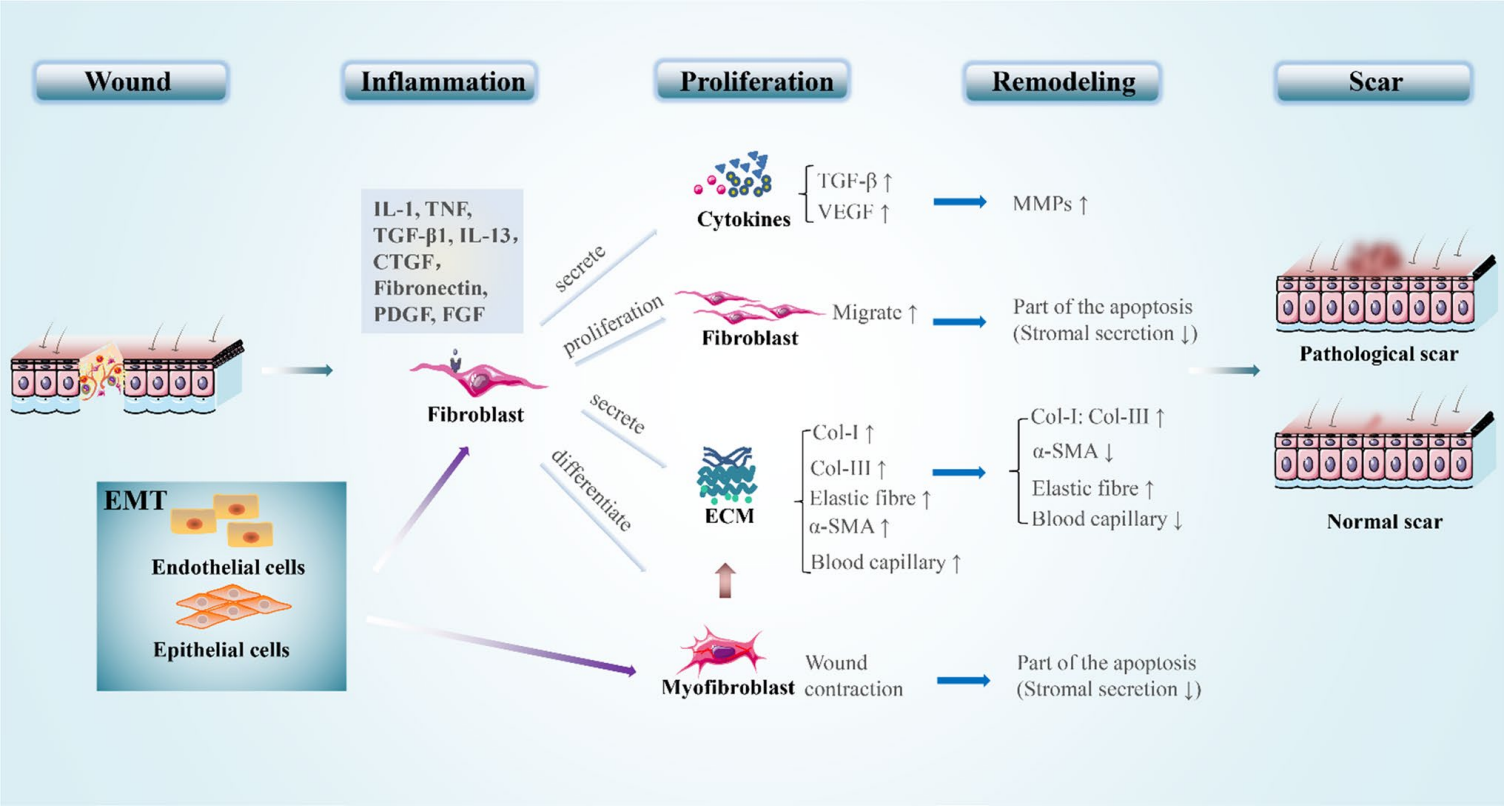
The role of fibroblasts and myofibroblasts in scar formation. (1) During the inflammatory phase, various cytokines and inflammatory factors stimulate fibroblasts to undergo phenotypic changes. (2) In the proliferative phase, fibroblasts produce large amounts of cytokines and extracellular components by secretory action, which cause ECM accumulation. Fibroblasts are transformed into myofibroblasts through differentiation, which cause wound contraction and further ECM accumulation. Meanwhile, the migratory of fibroblasts is enhanced. (3) During the remodeling phase, the extracellular component secreted by fibroblasts is reduced and the MMPs secreted by fibroblasts help the scar remodeling. Concurrently, fibroblasts and myofibroblasts are partially apoptotic, which contributed to the reduction of ECM. (4) EMT also has an important role in wound healing, during which epidermal cells and endothelial cells can differentiate into fibroblasts and myofibroblasts. “↑” and “↓” represent increase and decrease, respectively

In different tissues, myofibroblasts can be derived from regenerated epithelial cells and endothelial cells by means of epithelial-mesenchymal transformation (EMT) and endothelial-mesenchymal transformation [37]. Myofibroblasts are initially found in granulation tissue during the wound healing process, which have prominent endoplasmic reticulum for secretion and microfilaments for contraction [13, 38]. Myofibroblasts do not produce and contract the collagenous ECM simultaneously, and this process is mediated by different subtypes of myofibroblasts [38]. At the transcriptome level, cutaneous myofibroblasts are substantially different from pluripotent skin-derived precursors and fibroblasts in undamaged skin. Myofibroblasts maintain scar formation via epigenetic changes, such as DNA hypermethylation [13]. Although the α-SMA expression is not restricted to the myofibroblast [36], its expression is generally used as an indicator of myofibroblast phenotype [39]. It has been shown that the presence of this actin isotype not only enhances the contraction of myofibroblasts but also directs the activation of myofibroblasts in the intracellular mechanical feedback loop [36]. In contrast to the contractility of smooth muscle cells, the long-time contraction of myofibroblasts results in permanent tissue retraction [40]. This contraction in myofibroblasts partially explains the role of these cells in the formation and remodeling of excessive scarring, as seen in hypertrophic scarring and fibrotic tissue [26]. In addition, myofibroblasts promote cancer progression by stimulating microenvironment for epithelial tumor cells [41]. After the tissue integrity was restored, the activity of myofibroblast ceased and some cells apoptosis occurred [26, 42]. However, in hypertrophic scars (HTS), myofibroblasts continue to proliferate instead of apoptosis, because myofibroblasts are unresponsive to apoptosis-inducing factors. Moreover, myofibroblasts are also related to biomechanics. The increased skin tension results in up-regulation of genes related to matrix remodeling and down-regulation of genes related to apoptosis [37]. How to effectively control the formation, survival, and death of myofibroblast is one of the major challenges in wound treatment [36]. The role of fibroblasts and myofibroblasts in the process of scar formation is shown in Fig. 1.

## Characteristics of ADSCs and Exosomes

### ADSCs

**Fig. 2:**
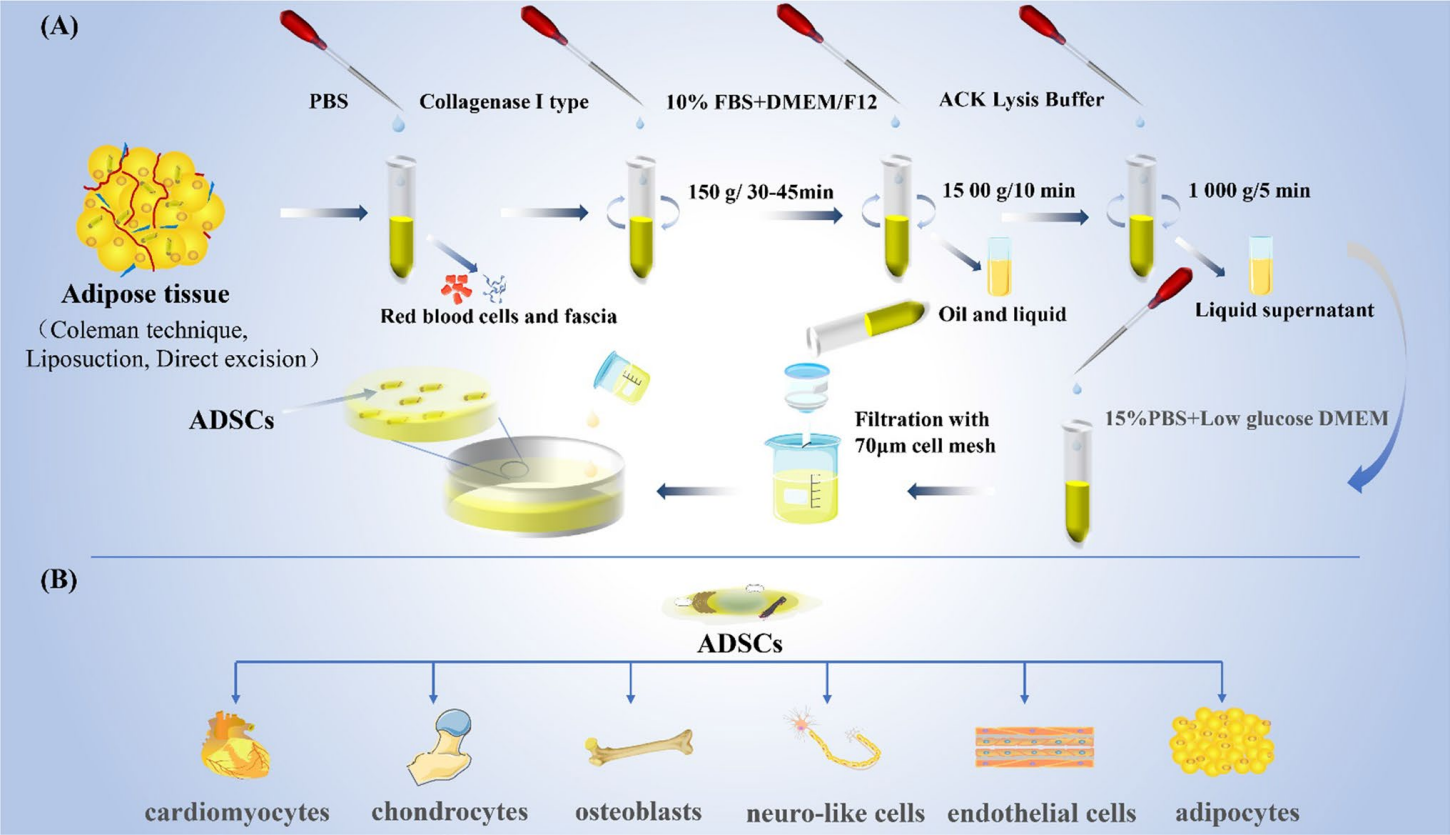
The extraction and differentiation of ADSCs. Adipose tissue should be collected from patients with no underlying disease, adverse lifestyle preferences, or a history of drugs that affect fat metabolism. 25 ml fat was taken and washed with PBS for 3 times to remove visible red blood cells. Then 0.075% type I collagenase of the same volume was added for 30–45 min digestion at 37 °C at 150 r/min, and DMEM/F12 medium containing 10%FBS was added to terminate digestion. After centrifugation, the top oil and intermediate clarifying solution are removed, and then the red blood cells are lysed with red blood cell lysate, followed by centrifugation to obtain a precipitate. The precipitate was resuspended in complete medium and filtered by 70 um cell sieve. After that, the resuspended solution was transferred to the culture dish for primary culture. ADSCs have the ability to differentiate into other mesoderm cells such as cardiomyocytes, endothelial cells, adipocytes, osteoblasts, chondrocytes, neuro-like cells

In vitro, ADSCs are spindle-shaped and lack intracellular lipid droplets, which can be seen in adipocytes [43]. ADSCs are derived from mesoderm and have the ability to differentiate into other mesoderm cells such as cardiomyocytes, endothelial cells, adipocytes, osteoblasts, chondrocytes, neuro-like cells, etc. (Fig. 2B) [44–46]. ADSCs can also regulate the surrounding microenvironment by continuously releasing extracellular components, such as miRNAs and growth factors, and have the effects of anti-apoptosis, anti-inflammatory, promoting angiogenesis, immune regulation, and anti-scar formation [47, 48]. ADSCs produce collagen more efficiently than other stem cells [43]. ADSCs possess stem cell-specific surface markers, such as CD90, CD105, CD73, CD44, and CD166, but the hematopoietic markers CD45 and CD34 are not expressed [49]. An ideal 3D biological scaffold can provide a suitable environment for ADSCs to promote their proliferation and maintain their differentiation ability [46]. Kim et al. observed that subcutaneous ADSCs had higher proliferation capacity and lipogenic differentiation capacity compared with those from the abdomen [50]. The same amount of ADSCs can be isolated regardless of the age of the donor [51]. On the contrary, other studies have shown that the total cell production of ADSCs can be reduced in the influence of age [52]. In animal experiments, ADSCs have a higher proliferation capacity in young animals [51]. The ability of proliferation, differentiation, paracrine, and anti-apoptosis of ADSCs varies with the sex of the donor. The regeneration potential of ADSCs decreased in patients with chronic diseases. The risk of cancer induced by ADSCs transplantation has not been completely ruled out [51].

ADSCs have a large storage in adipose tissue and can be obtained with less invasive procedures, without ethical limitations [18, 53]. Although bone marrow mesenchymal stem cells (BMSCs) or umbilical cord mesenchymal stem cells (UCMSCs) have shown some therapeutic advances in the treatment of ulcers, scars, and burns, the proliferation, differentiation and paracrine abilities of ADSCs have demonstrated their advantages in a wide range of applications in this field [54]. In addition, compared with BMSCs, ADSCs are more genetically and morphologically stable in long-term culture with higher proliferation activity [55]; showed more osteogenic differentiation in 3D scaffolds [56]; are more suitable for survival in anoxic environment and show advantages in regulating inflammation [57]; have shown great anti-inflammation, anti-phagocytosis, anti-apoptosis and cell viability in the aspect of anti-atherosclerosis [58]; showed better regeneration in tendon injury, with a significant increase in the number of myotubes and a significant decrease in collagen deposition [59]; showed stronger ability of neuronal differentiation and neurotrophic factor secretion in cell transplantation therapy for nervous system injury [60]. Meanwhile, ADSCs showed stronger osteogenic ability than dental pulp stem cells [61]. Furthermore, ADSCs can differentiate into three developmental dermal cell types (endoderm, mesoderm and ectoderm) [62]. These advantages make ADSCs the most attractive source of MSCs for regenerative medicine. The advantages and disadvantages of human stem cells are shown in Table 1 [63].

**Table 1:** Advantages and disadvantages of the stems cells found in the human body

Name of Cell Type	Definition	Advantages	Disadvantages
Adipose tissue-derived stem cells	Cells derived from adipose tissue	High multipotent potential and no ethical issues; Easy access with minimal damage	Potentially tumorigenic; repair tissue mechanism not fully understood
Bone marrow stem cells	A stem cell found in the bone marrow that can form erythrocytes, leukocytes, and platelets	Widely used in research	Low acquisition rate; take on fused donor/recipient characteristic; easy to contaminate in culture
Embryonic stem cell	Pluripotent stem cell derived from embryo	Great pluripotent and regenerative properties	With ethical limitations; potential immune rejection during transplant to recipient
Induced pluripotent stem cells	A somatic (adult) cell that is induced to show embryonic cell-like properties	Low immune response to the recipient; ability to differentiate into cardiomyocytes	Poor purity; difficulty in controlling unwanted heterogenous cell differentiation
Dental pulp stem cells	Stem cells derived from dental pulp	Polydifferentiated ability; no ethical restrictions; low immunogenicity	Low acquisition rate; compared with ADSC, the osteogenic capacity is low

Current techniques for obtaining adipose tissue used to extract ADSCs include the coleman technique, liposuction, and direct excision [46]. The extraction process is shown in Fig. 2A [64]. ADSCs can be stored in conventional cryopreservation media, including 90% FBS and 10% Dimethyl sulfoxide (DMSO) [46].

### Exosomes

Exosomes are a type of extracellular vesicle enclosed by lipid membranes between 40 nm and100 nm in diameter. Exosomes are formed in cells through the mechanism of endocytosis [65]. Exosomes were cup-shaped with a density of 1.13–1.19 g/mL [64]. The goblet shape can be used to distinguish between cell-derived vesicles and particles of similar size [66]. There are special markers on the membrane surface of exosomes, such as membrane-binding proteins CD81, CD9, CD63, MHC-I, heat shock proteins 73, 90, etc. Exosomes contain a variety of microRNA, proteins, cytokines, lipids, and unedited RNA [67]. Xing et al. analyzed the mouse ADSC-Exos and identified a total of 1185 proteome. The pathway analysis showed that most proteins were involved in the metabolic pathway, adhesion plaques, regulation of actin skeleton, and microbial metabolism [68]. Therefore, exosomes play an important role in regulating different physiological and pathological processes and participate in inter-cell signal transmission at different distances, such as substance transmission, signal transmission, cell survival, apoptosis, and cell proliferation. In addition, some studies have shown that miRNA in exosomes can regulate the expression of target genes in recipient cells [24, 67]. Meanwhile, exosomes have the following characteristics: source cells characteristics, long-time activity, easy to transport, low immunogenicity, easy to control the concentration and the contents change with the microenvironment [69–72].

MSC exosomes (MSC-Exos), like exosomes derived from other cells (tumor cells, immune cells, nerve cells, etc.), can perform many functions as intercellular shuttles. MSC-Exos have the characteristics of maintaining tissue homeostasis and responding to the external environment. In addition, they can potentially restore normal tissue function by providing catalytic active enzymes, and when tissue damage occurs, MSC-Exos are endocytosed by damaged tissues, restoring normal cell function. Meanwhile, MSC-Exos have good tolerance, long life and better bioavailability [73]. Although bone marrow mesenchymal stem cell exosomes and umbilical cord mesenchymal stem cell exosomes play a role in promoting wound healing and alleviating scars [74], their shortcomings of difficult access and ethical limitations limit their use. Therefore, ADSC-Exos has attracted increasing attention.

**Fig. 3:**
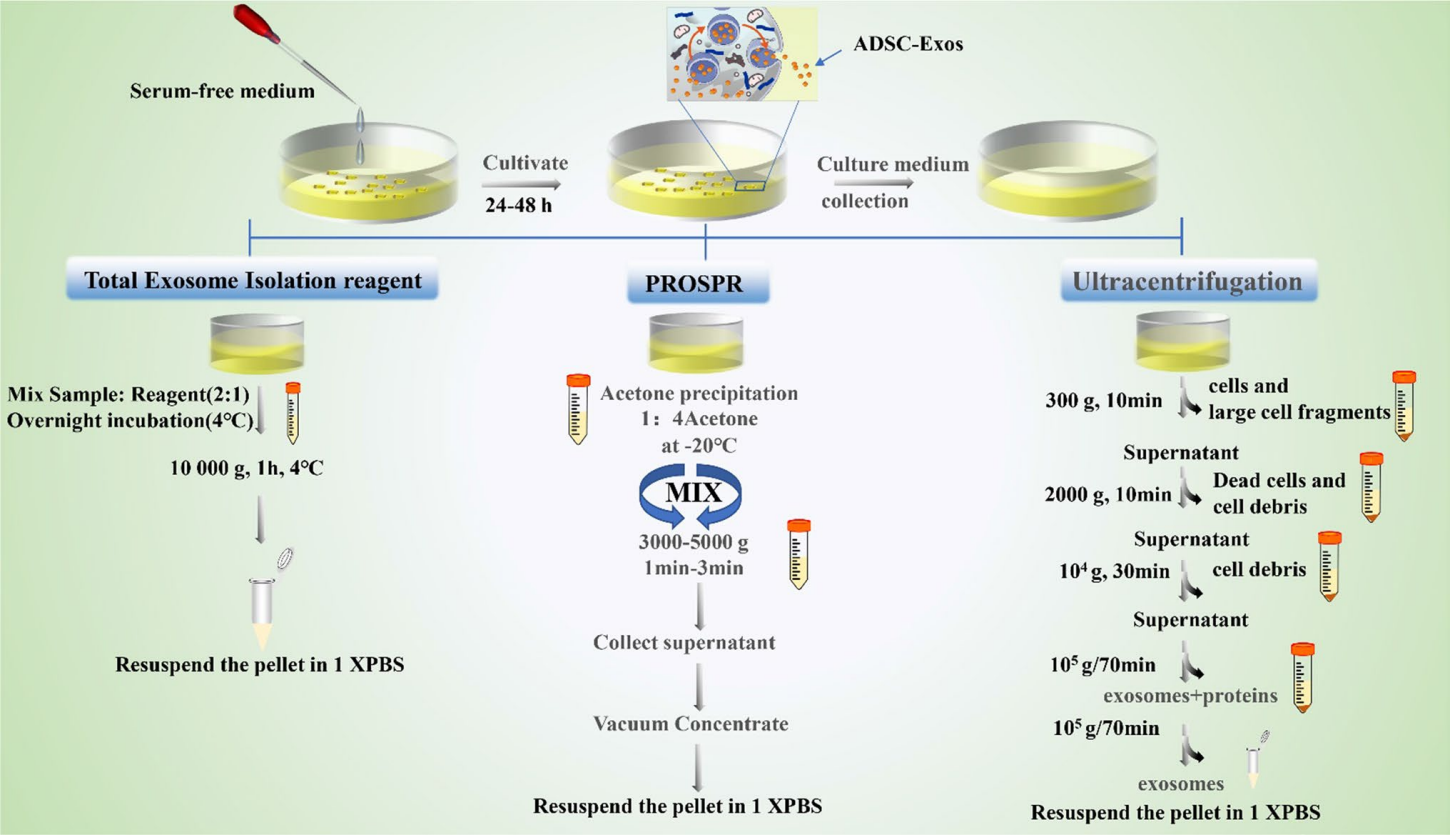
The three isolation techniques used in isolation of exosomes from serum-free conditioned media. (1) TEI the method where the serum-free conditioned media was taken and mixed with the reagent in 2:1 ratio, vortexed properly and incubated overnight at 4 °C. The exosomes were pelleted down at 10000 g for 60 min at 4 °C and were resuspended in 100 μl of 1 × PBS for further studies. (2) PROSPR is a technique that the conditioned media was mixed with ice-cold acetone in a ratio of 1:4 and vorutexed, then centrifuged at 3000 g for 2 min. The supernatant was collected and concentrated in a vacuum concentrator in vacuum-alcohol mode. The concentrated crystals were resuspended in 100 μl 1 × PBS. (3) The supernatant collected from this stage was centrifuged at 100000 g twice for 70 min each time to separate exosomes from the precipitation in the final step. The first hypervelocity rotation was to remove larger vesicles. The supernatant was discarded, and the precipitate was washed with 1× PBS in the second rotation. The resuspension of the precipitation is the same as before

The extraction methods of ADSC-Exos include ultracentrifugation, Protein organic solvent precipitation (PROSPR), and total exosome isolation reagent (TEI) [75]. In the process of obtine exosomes, FBS is prohibited from adding cell culture medium. The specific steps of ADSC-Exos extraction are shown in Fig. 3 [75]. Micromorphology under electron microscopy is the gold standard for the identification of exosomes. Besides, it can also be identified by specific markers on the surface of exosomes [67].

## The Effect of ADSCs on the Behavior of Fibroblasts and Myofibroblasts during the Process from Wound to Scar

In the early stages, ADSCs facilitate the proliferation of skin fibroblasts, which in turn promote wound healing and collagen production [76]. However, in the late stage, ADSCs inhibit fibroblast proliferation and collagen synthesis [77]. Hence, ADSCs play different roles in different stages between wound healing and scar stage. Recent studies have shown that ADSC-CM and ADSC-Exos are the main factors for ADSCs to exert its biological effects [78]. So, we summarized the roles of ADSC-CM and ADSC-Exos of ADSCs in the process of wound and scar stage.

### Wound Stage

#### ADSC-Cm

In the experiment of Lee et al., they collected ADSCs suspension (ADSC-CM) and cultured fibroblast together. They found that ADSC-CM promoted the proliferation of fibroblasts and the contraction of collagen lattice in fibroblasts. Meanwhile, the expression of Col-I gene in fibroblasts was up-regulated by ADSC-CM [79]. Different culture conditions of ADSCs have different effects on the biological characteristics of fibroblasts. Compared with 2D medium, ADSCs cultured in 3D medium significantly promote fibroblasts’ migration with a high expression of actin [80]. In the experiment of Yu et al., they made wafer with ADSCs and L-ascorbate 2-phosphate. They found that TGF-β1 and α-SMA were down-regulated in the ADSC-CM-cultured fibroblasts, which proved that ADSCs wafer had the effect of anti-scar formation and optimize the quality of new skin during wound healing [81]. Shukla et al. observed that ADSCs reversed radiation-induced hypermigration of dermal fibroblasts [82]. In the experiment of Woo-Chan Son et al., they treated fibroblasts with ultraviolet radiation and then treated them with ADSC-CM. They found that MMP-1 expression was significantly increased, which was conducive to scar remodeling [83]. Zhao et al. found that EGF, PDGF-AA, VEGF, and basic fibroblast growth factor (bFGF) were found to be in high concentrations in ADSC-CM, and VEGF, bFGF, and PDGF-AA significantly stimulated the migration of skin fibroblasts, and bFGF and EGF can significantly stimulate the proliferation of vascular smooth muscle cells [84].

#### ADSC-Exos

In the experiment of Choi et al., they cultured ADSC-Exos with human skin fibroblasts in vitro and found that the expression of genes associated with skin regeneration (CD34, Col-I, elastin, and keratinocytes) was increased in a dose-dependent manner. At the same time, the proliferation rate of human dermal fibroblasts (HDFs) also increased, especially in the S phase of these cells [72]. Wang et al. investigated the effect of ADSC-Exos on the expression of ECM-related genes in skin fibroblasts. The mRNA expression levels of α-SMA and Col-I A1 were reduced, and TGF-3, Col-III A1, MMP1, and MMP3 were increased, while TIMP-1 and TGF-1 remained basically unchanged. The level of protein expression is similar to that of the mRNA. It can be seen that ADSC-Exos can regulate the ratio of fibroblast Col-III to Col-I, TGF-3 to TGF-1, and MMP3 to TIMP-1, as well as regulate fibroblast differentiation to affect ECM reconstruction, thereby alleviating scar [85]. Besides, ADSC-Exos can transfer fibroblasts to an endogenous state and inhibit their differentiation [37].

The migration and proliferation of fibroblasts are also affected by ADSC-Exos in a dose-dependent manner [23]. Choi et al. showed that ADSC-Exos contained miRNAs that inhibited genes including NPM1, PDCD4, CCL5, and NUP62, thus contributing to the proliferation of skin fibroblasts [72]. Exosomes also promote the migration and proliferation of fibroblasts by promoting the mRNA expression of N-cadherin, cyclin-1, and PCNA [23]. Cooper et al. found that ADSC-Exos could increase dermal fibroblast migration and accelerate ischemic wound healing by releasing lncRNA MALAT1 (metastasis-associated lung adenocarcinoma transcript 1) [86]. Qian et al. found that ADSC-Exos could promote the proliferation of fibroblasts via lncRNA H19/ Mir-19b /SOX9 Axis, thus speeding up wound healing [87]. Akt is one of the pathways that ADSC-Exos enhances the proliferation and migration of fibroblasts, which is independent of miRNA-205 [88]. Zhang et al. experim reduced ent also indicated that PI3K/Akt is a way for ADSC-Exos to regulate fibroblast. In a medium containing ADSC-Exos, if fibroblasts are pretreated with PI3K inhibitors Ly294002, the levels of cells proliferation, phosphorylation of Akt, Col-I and Col-III will be [89]. Wang et al. explained that ADSC-Exos may increase the MMP3 level of fibroblasts in the manner of ERK/MAPK signaling pathway. ADSC-Exos also induced more nuclear translocations of P-ERK in fibroblasts. Besides, they observed that ADSC-Exos increased the expression of downstream genes in the ERK/MAPK pathway (c-Jun, c-Fos), while the increased expression was almost completely eliminated by the P-ERK-specific inhibitor U0126 [85]. ADSC-Exos can transport functional cytoskeleton proteins (such as vimentin), which can act as promoters of fibroblast proliferation, migration, and ECM secretion [90]. ADSC-Exos also enhances the migration of human skin fibroblasts by lncRNA MALAT1(metastasis-associated lung adenocarcinoma transcript 1) [86].

### Scarring Stage

#### ADSC-Cm

It has been reported that ADSC-CM can reduce the expression of Col-I, Col-III and β-smooth muscle actin (β-SMA) in vitro, which are caused by the action of anti-fibrotic factors in ADSC-CM, thereby reducing collagen deposition and scar formation [91]. ADSC-CM significantly inhibits the proliferation and migration of hypertrophic scar fibroblasts, then lowers the expression level of ECM molecules in cells [78]. The result of Ma et al. (2020) showed that ADSCs could reduce the activity of fibroblasts, fibrosis molecular expression, and TIMP-1 in hypertrophic scar by secreting hepatocyte growth factor (HGF), while significantly increase the expression of MMPs [92]. In addition, the P-P38 protein level of hypertrophic scar fibroblasts cultured with ADSC-CM was down-regulated in a concentration-dependent manner, and collagen was arranged more orderly [93]. In the hypertrophic scar model of rabbit ear, Chu et al. found that ADSCs could significantly increase the expression of decorin (DCN) in fibroblasts. The core protein is the most critical protein in DCN, which can transmit different biological signals and resist scar formation [94]. The expression of p53 in ADSCs is associated with hypertrophic scar [95]. If the p53 gene of MSCs is knocked out, the production of NO will be increased and the ability to inhibit the proliferation of fibroblasts will be reduced [96]. ADSCs inhibit TGF-β1-induced differentiation of fibroblasts in adult skin and TGF-β1-induced contraction of keloid through the paracrine way. Furthermore, ADSCs down-regulate intracellular signaling pathway related molecules (such as p-Smad2, p-Smad3, p-STAT3, and p-ERK) and proteins, which are also important ways to inhibit hypertrophic scar [95]. In the experiment of Li et al. (2016), ADSC-CM can reduce collagen deposition and scar formation in vitro, ex vitro, and in vivo through the p38/MAPK signaling pathway [93]. Wang et al. cultured keloid fibroblasts with ADSC-CM, and they found that ADSC-CM decreased the expression of extracellular matrix related genes, inhibited cell proliferation and migration, and reduced CD31^+^/CD34^+^ blood vessels, collagen deposition and TIMP [97].

#### ADSC-Exos

**Fig. 4:**
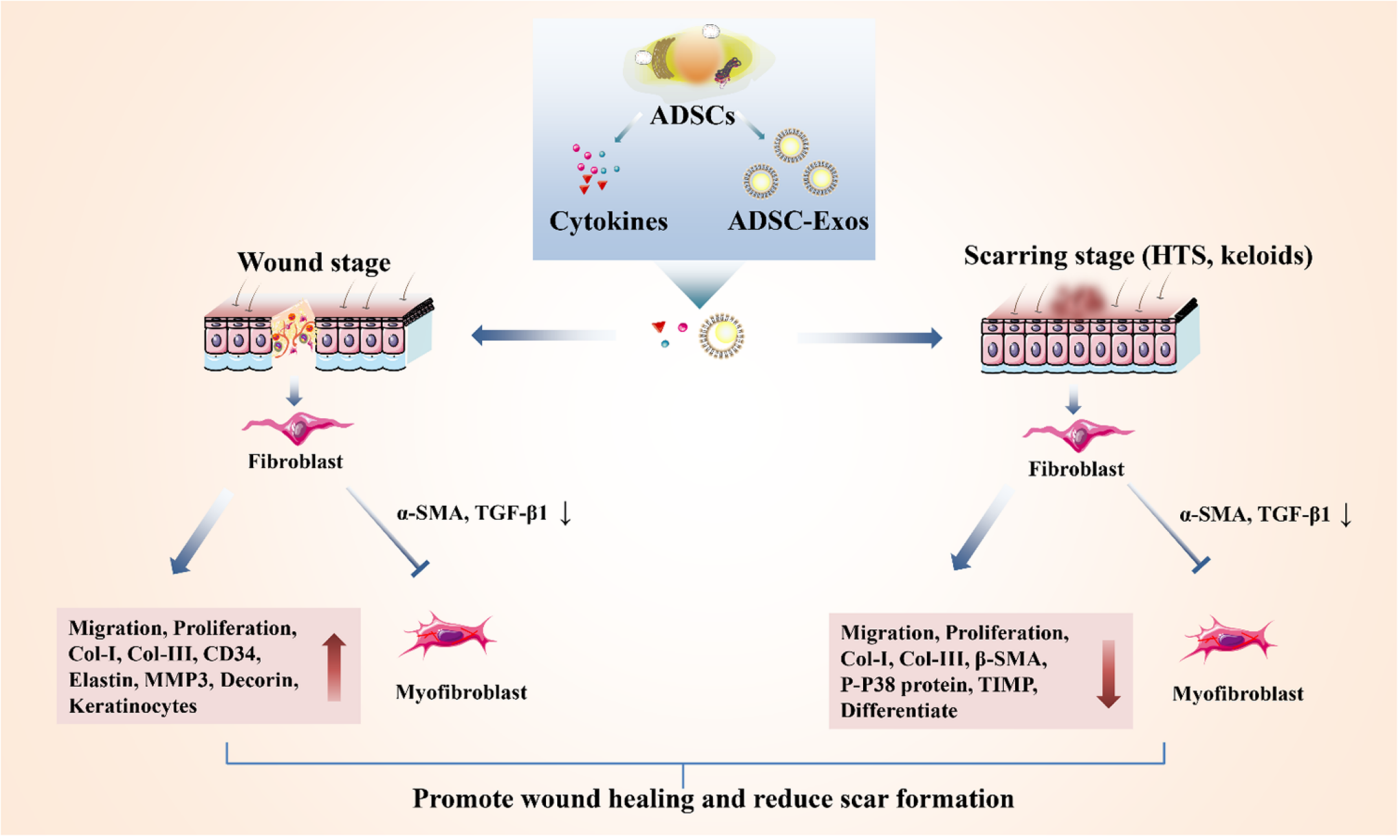
The effect of ADSCs on the biological characteristics of fibroblasts and myofibroblasts during the process from wound to scar. The process of ADSCs in preventing scar formation is complex and has different roles in the wound stage and scar formation stage. In the wound stage, ADSCs and ADSCs-Exos increased the migration, proliferation, Col-I, Col-III, CD34, elastin, MMP3, decorin, keratinocyte of fibroblasts. In the scar stage, ADSCs and ADSC-Exos decreased the migration, proliferation, Col-I, Col-III, β-SMA, P-P38 protein, TIMP, differentiate of fibroblasts. However, they reduced α-SMA and TGF-β1 and inhibited the transformation of fibroblasts into myofibroblasts in both the wound and scar stage. “↑” and “↓” represent increase and decrease, respectively

ADSC-Exos can effectively inhibit the proliferation and migration of hypertrophic scar fibroblasts, reduce the expression of Col-1,Co-III, α-SMA, IL-17RA and p-Smad2/p-Smad3, and increase the level of SIP1 in fibroblasts. Mice treated with ADSC-Exos showed faster wound healing and less collagen deposition [98]. miR-192–5p is also the regulatory mode of ADSCs to reduce the fibrosis level of hypertrophic scar [98]. The effects of ADSCs on the biological characteristics of fibroblasts and myofibroblasts during the process from wound to scar are shown in Fig. 4.

## ADSCs and ADSC-Exos Indirectly Regulate Fibroblasts and Myofibroblasts by Promoting Angiogenesis and Inhibiting Inflammation, Thereby Reducing Scar Formation

### ADSCs and ADSC-Exos Promote Angiogenesis

Lynam et al. observed that moderate hypoxia (5% O2) and malnutrition (5% FCS) increase the level of fibroblasts and collagen, however, severe hypoxia (0.5% O2) and malnutrition (0.5% FCS) reduces the production of collagen, the cell vitality, and induce cell apoptosis [99]. The continuous overexpression of multiple cytokines by fibroblasts under the stimulation of vascular endothelial growth factor (VEGF) leads to excessive inflammation and collagen production. Excessive collagen deposition can mechanically crush the microvessels, resulting in occlusion of the microvessels and hypoxia at the damaged site, which further induce collagen formation [9]. Chen et al. found that the hyperactive glycolytic fibroblast population is the main factor for ECM deposition during skin trauma, suggesting that glycolytic diversity is closely related to the heterogeneity of fibroblasts. Hyperactive glycolysis may be a functional phenotype in patients with fibrosis [10]. The enhancement of cellular glycolysis is usually caused by insufficient oxygen supply to the tissues, but the oxygen supply depends on the blood supply of the tissues. Therefore, blood supply is also a factor affecting fibroblast phenotype.

Current studies have shown that ADSCs and ADSC-Exos play an important role in angiogenesis. ADSCs have great potential to release angiogenic factors either by injection or stent delivery [51]. Luo et al. studied the biaxially secretory effect between microderms and ADSCs, and the results showed that the combination of microderms and ADSCs can upregulate cytokines, such as VEGF, IL-6, HGF, and EGF [100]. ADSC-Exos is comparable in angiogenesis to ADSCs [101]. Microenvironmental changes can affect the angiogenesis of ADSC-Exos. According to the experiments of Han et al., in terms of angiogenesis, the ability of hypoxic-treated ADSC-Exos to form capillary networks is higher than that of non-hypoxic-treated ADSC-Exos [102]. In the experiment of Bail et al., ADSC-Exos pretreated with H_2_O_2_ could promote angiogenesis [103]. Liang et al. showed that ADSC-Exos can transfer miR125a to endothelial cells and promote angiogenesis by inhibiting DLL4 [104]. In addition, ADSC-Exos promotes angiogenesis by delivering miR378a-3p [105]. In some cases ADSCs can induce wound healing, but in some cases ADSCs can cause and aggravate hyperplastic scarring such as in some stages of acute burns, and this may be due to the excessive angiogenesis and granulation tissue [9].

### Effects of ADSCs and ADSC-Exos on Inflammation

During wound healing, macrophages, lymphocytes, and other inflammatory cells release various factors that induce fibroblast proliferation [95]. Therefore, inflammation is an important factor affecting fibroblasts’ activity. Roh et al. reported that human MSCs were implanted on the polymer scaffold, then put the scaffold in immunodeficient mouse, and the cells could not be detected within a few days. Instead, the scaffolds were initially refilled by mouse monocytes, followed by refilled by smooth muscle cells and endothelial cells. Therefore, the authors first hypothesized that MSCs secrete a large amount of monocyte chemotactic protein-1, thus increasing the recruitment of early monocytes in the mouse. These findings suggest that tissue regeneration occurs through an inflammatory process and not just through cell recovery [106]. Previous studies have shown that ADSC-Exos can effectively protect tissues and organs from ischemia-reperfusion injury by regulating inflammatory and oxidative signal transduction axes [107]. Therefore, ADSCs and ADSC-Exos can indirectly regulate fibroblasts and myofibroblasts through the effects on inflammatory response in the process of wound healing.

**Fig. 5:**
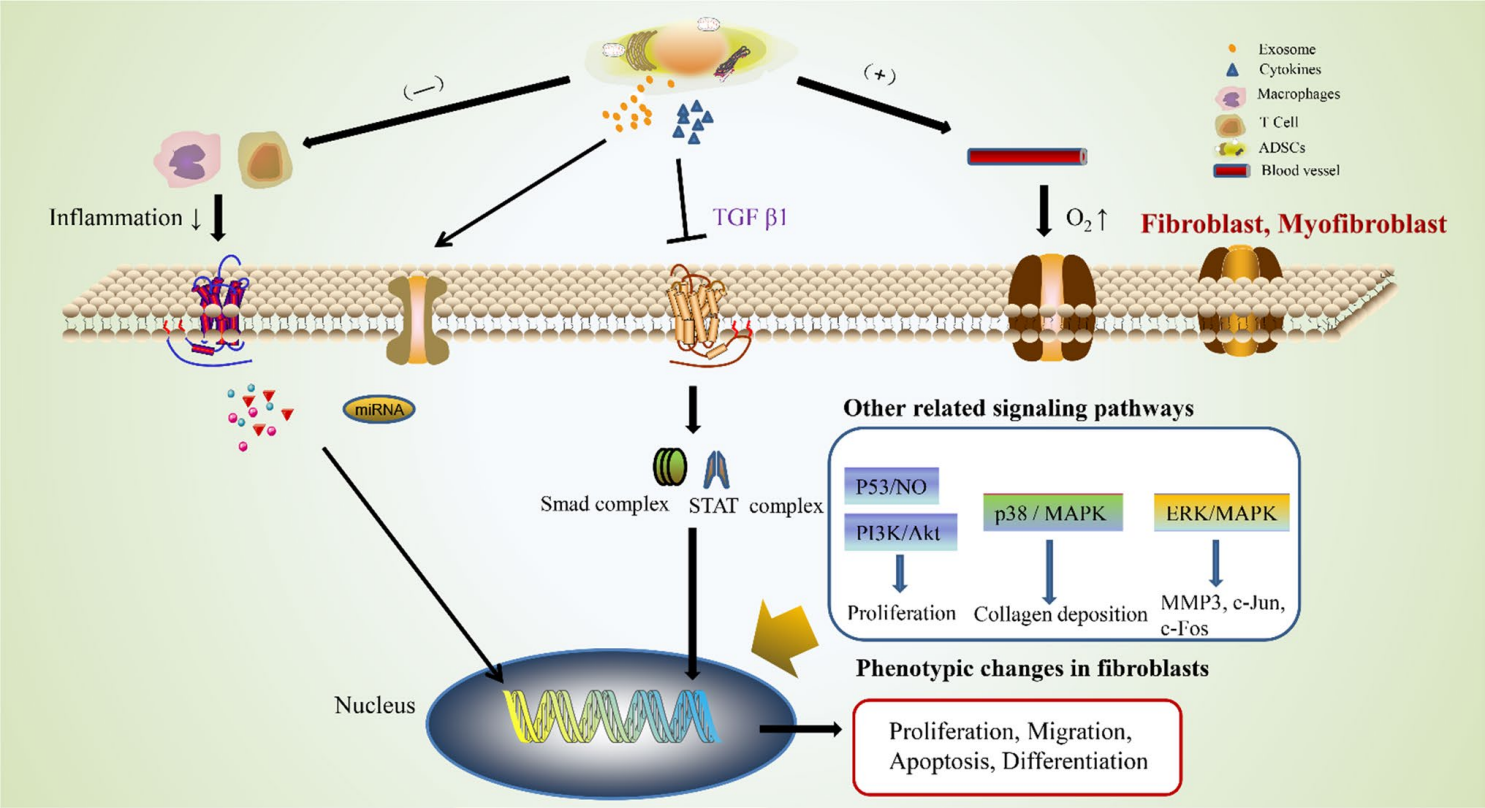
The mechanism of effect of ADSCs and ADSC-Exos on the biological characteristics of fibroblasts and myofibroblasts. Four main mechanisms are summarized: (1) ADSCs and ADSC-Exos can inhibit the inflammatory response of macrophages and T cells, thereby attenuating the release of inflammatory factors, which in turn attenuates the response of fibroblasts and myofibroblasts to inflammation. (2) miRNA secreted by ADSCs and miRNA containde by ADSC-Exos can enter fibroblasts and myofibroblasts, then directly participate in the transcription and translation of genes. (3) ADSCs secrete various cytokines, which are involved in the regulation of fibroblasts and myofibroblasts through different signaling pathways. (4) ADSCs and ADSC-Exos promote angiogenesis, which enhance oxygen uptake by fibroblasts and myofibroblasts, then optimize the metabolism of fibroblasts and myofibroblasts. “­” and “+” stand for inhibition and promotion, respectively

ADSCs have the abitily to exchange cytoplasmic components bidirectional with primary T lymphocytes [108]. ADSC-Exos is similar to its adipose stem cell source in its up-regulation of early inflammation [101]. ADSC-Exos can coordinate the role of CD4^+^ T cells in the immune system, such as coordinating the balance between various subsets of CD4^+^ T cells [109]. In vitro, ADSC-Exos demonstrated the ability to inhibit T cell differentiation, reduce T cell proliferation, and stimulate the release of interferon-γ [110]. Macrophages play a role in coordinating the microenvironment during wound healing. From the early stage to pathological scar formation, the polarization of macrophages showed the temporal and spatial diversity of M1 and M2 macrophages. The increased number of M2 cells is closely related to the sensitivity of the pathogenesis of abnormal scar [111]. Inflammatory cytokines can increase the immunosuppressive and anti-inflammatory abilities of ADSC-Exos, which have the ability to transform macrophages from M1 phenotype to M2 phenotype by regulating macrophage polarization through miRNA shuttles [112]. ADSC-Exos also upregulate the expression of M2 macrophage markers to regulate macrophage polarization [113], and increase the mRNA levels of M2-associated arginase-1 and IL (interleukin)-10 [114]. In addition, ADSC-Exos activated arginase-1 and transcriptional activator 3 (STAT-3), which induced polarization of macrophages to an anti-inflammatory M2, and significantly inhibited lipopolysaccharide (LPS) and IFN-γ-stimulated macrophage inflammatory response [114]. In the rat model of intestinal perforation causing systemic inflammatory response, the survival rate of rats treated with ADSC-Exos was significantly increased and the rats showed significant low inflammatory response [115]. The mechanism of effect of ADSCs and ADSC-Exos on fibroblasts is summarized in Fig. 5.

## Problems of ADSCs and ADSC-Exos in Anti-Scar Application

Although there are many studies on ADSCs in wound healing, we still have little understanding of their mechanism of action [95]. So, we still have a lot of work to do to understand the mechanisms as far as possible. The contents of exosomes depend on the cells they come from and the physiological conditions of the cells [116]. How to identify the specific contents of various exosomes and how to effectively control and regulate the contents of exosomes are part of the problems we need to solve.

Meanwhile, due to high clearance rate and short half-life, the application of exosome in wound healing remains a challenge. Besides, their function may be impaired, as regeneration usually takes a long time and the viability of free exosomes is not maintained, which is also an issue we need to address. To solve the above problems, the combination of ADSCs and ADSC-Exos with biomaterials is also a research hotspot. Wang et al. manufactured an injectable adhesive, heat-sensitive multifunctional polysaccharide dressing (FEP), which has sustained pH response to exosome release, and promotes angiogenesis and diabetic wound healing [117]. In order to address the poor organ-targeting capability of exosomes in MSCs, Li et al. labeled exosomes with oxidized nanoparticles (Exo + NPs) and injected Exo + NPs into the body under magnetic guidance. This method significantly increases the amount of Exo + NPs accumulated at the site of injury. These accumulated Exo + NP reduce scar formation and increase the expression of CK19, PCNA, and collagen in the body [118].

ADSC-Exos itself has the function of the carrier, and can also be used as a component of well-designed biomedical materials. ADSC-Exos can be used as a stable and effective carrier to load specific proteins, lipids, and genetic materials, and preferentially transport them to target tissues or organs due to its inherent homing ability or targeting ability of artificial modification [119, 120]. How to use the delivery function of exosomes still needs further study. In the experiment of Bolandi et al., they introduced miR-10a into exosomes by means of electric shock to regulate CD4^+^ T cell differentiation [109].

Precision therapy is the invariable direction of basic and clinical research. Fibroblasts and myofibroblasts play different roles in different stages of wound healing. Therefore, it is necessary to clarify the regulatory effects of ADSCs and ADSC-Exos on fibroblasts and myofibroblasts at different stages of scar formation. Unfortunately, the role of ADSCs and ADSC-Exos in the different stages of scar formation has not been studied. Depending on the physiological or pathological status of the host tissue, fibroblasts show different shapes and sizes, and represent heterogeneous populations of cells with different characteristics that remain largely undefined. During the wound repair process of skin tissue, fibroblasts show considerable functional differences, for example, there is little scar formation during wound remodeling in the mouth, while there is much scar tissue deposition in skin wounds [121]. Therefore, it is necessary to study the effects of ADSCs and ADSC-Exos on fibroblasts from different host tissue.

## Conclusion & Expectation

In conclusion, it can be known that ADSCs and ADSC-Exos can regulate fibroblasts and myofibroblasts in various ways. Therefore, ADSCs and ADSC-Exos have an enormous potential in clinical application of anti-scar therapy. With the further study of ADSCs and ADSC-Exos as well as their relationship with biomaterials, the application of ADSCs and ADSC-Exos in scar treatment will be realized in the future.

## Data Availability

Not applicable.
